# Time-Restricted Feeding and Caloric Restriction Impact on Spontaneous Neoplasms in Female Mice

**DOI:** 10.1093/geroni/igaa057.420

**Published:** 2020-12-16

**Authors:** Annamaria Rudderow, Eleonora Duregon, Michel Bernier, Rafael de Cabo

**Affiliations:** National Institute on Aging, Bethesda, Maryland, United States

## Abstract

In older humans, multiple chronic diseases and increased life expectancy impose a disproportionate socioeconomic burden. Dietary interventions are valuable strategies for promoting healthy aging. Caloric restriction (CR) without malnutrition is a robust intervention able to delay disease onset and increase survival in model organisms. However, the impracticability of chronic CR outweighs the potential long-term benefits in humans. Time-restricted feeding (TRF), i.e. the limitation in the timing of food intake without necessarily reducing caloric intake, can protect against metabolic disorders through the synchronization of the circadian rhythm. This study compares whether limiting access to ad libitum (AL) food for a few hours per day mimics the beneficial effects of a CR diet. A large cohort of C57BL/6J female mice (n=250) was distributed into five feeding paradigms at midlife: AL, TRF for 8 hours, TRF for 4 hours, 20% CR and 20% CR fed twice a day (CRx2). Pathological analyses at death reveal a shift in fatal neoplasms toward an older age in TRF8 mice. AL mice had the highest prevalence of tumors (93%) and TRF4 had the lowest (77%). The highest tumor burden was observed in AL mice while CRx2 animals had the lowest number of neoplasms. Histiocytic sarcoma and lymphoma were the most represented malignancies, with CR mice exhibiting the highest rate of histiocytic sarcoma (75%) and the lowest rate of lymphoma (10%). These results indicate that time- and calorie-restricted feeding regimens can slow down malignant neoplasm progression and extend health span in female mice, even when started in adulthood.

